# Deep phenotyping ILCs and T cells: A comparative analysis protocol for conventional and spectral flow cytometry

**DOI:** 10.1016/j.xpro.2026.104669

**Published:** 2026-07-16

**Authors:** Jadie Acklam, Sukhveer K. Mann, Karen G. Hogg, Richard Leach, Andy C. Rawstron, Ruth M. De Tute, Catherine Cargo, Sinisa Savic, Ian S. Hitchcock, Jillian L. Barlow

**Affiliations:** 1 Centre for Blood Research, York Biomedical Research Institute, Department of Biology, University of York, Heslington, York YO10 5DD, UK; 2 NIHR, Leeds-York Biomedical Research Centre, Leeds, UK; 3 Department of Biology, Bioscience Technology Facility, University of York, York YO10 5DD, UK; 4 Haematological Malignancy Diagnostic Service, St James’s University Hospital, Leeds, UK; 5 Institute of Rheumatic and Musculoskeletal Medicine, University of Leeds, Leeds, UK

**Keywords:** Health Sciences, Immunology, NMGN Focused Collection

## Abstract

Here, we present a protocol for identifying human innate lymphoid cells (ILCs) and T cells using a 19-plex flow cytometry panel. We describe steps for titrating antibodies, preparing single-stain controls and samples, and staining cells for human peripheral blood and bone marrow samples. We then detail procedures for transferring a flow cytometry panel optimized for conventional cytometry to spectral flow cytometry analysis. This protocol facilitates correlation with murine models, maximizes measurable parameters, resolves complex fluorophore combinations, and increases panel flexibility.

## Highlights

• Deep phenotyping flow cytometry panel for human ILC and T cell subsets
• Considerations for the initial design of deep phenotyping flow cytometry panels
• Steps for sequential antibody staining of chemokine receptors
• Comparison of conventional and spectral cytometer analysis, including light-loss imaging

## Abstract (editor highlights)

Publisher’s note: Undertaking any experimental protocol requires adherence to local institutional guidelines for laboratory safety and ethics.

## Graphical abstract

**Figure:**
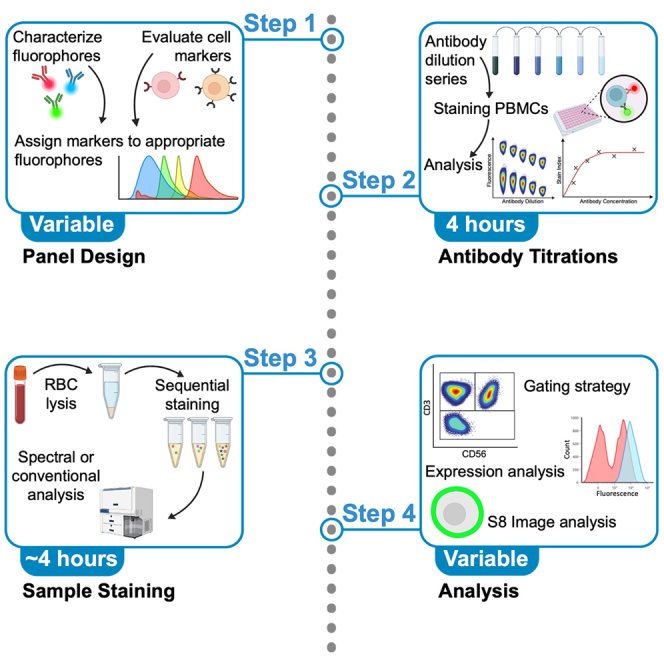

## Before you begin

We outline the processing and analysis of human peripheral blood and bone marrow samples using a multiplex flow cytometry panel to evaluate ILC and T cell subsets. Although this protocol has been specifically optimized for characterizing human samples, the overall approach can also be used for evaluating murine tissues. In addition, it provides a protocol that can be easily adapted between conventional and spectral flow cytometry. Spectral cytometry is a newer technique that allows collection of excited light from fluorescent proteins across the light spectrum, rather than from distinct wavelength ranges, to potentially greatly increase resolution and allow fluorescent proteins with greater spectral overlap to be combined together^1^ All personnel undertaking these experiments must have received appropriate training for human biological materials and work with relevant ethical and regulatory requirements.

### Cytometer and fluorophore characterization

**Figure 1:**
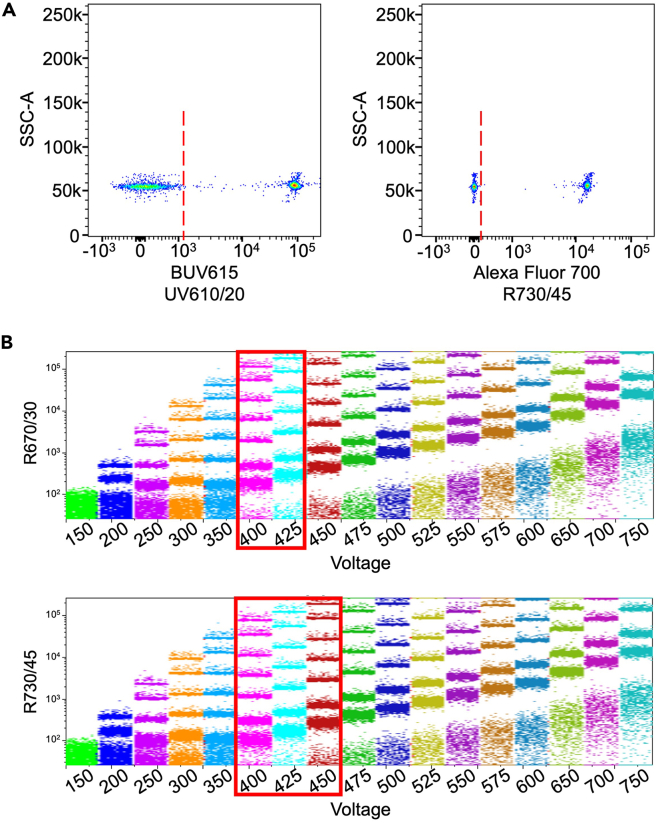
Characterization of fluorophores and cytometer instrument (A) Single-stained beads analyzed on the FACSymphony A3 cytometer, illustrating a fluorophore (Brilliant Ultraviolet, BUV615) with relatively large negative spread and a bright positive population, and a fluorophore (Alexa Fluor 700) with reduced negative spread and a dimmer positive population. Red dashed lines indicate limit of detection; beyond this line, events can more confidently be classified as positive. Analysis performed using BD FlowJo 10.10.0 software. (B) Eight-peak calibration beads were acquired on the BD FACSymphony A3 cytometer during incremental increases in detector voltage. Two representative plots. The red box indicates the optimal voltage range where all eight bead intensities are visible. Analysis performed using Beckman Coulter Kaluza 2.2 software.

Flow cytometers vary in their laser configurations, detector sensitivity, optical filters, and overall instrument setup, leading to variations in fluorophore behavior. In this protocol we use Becton Dickinson (BD) instrumentation, and although applicable to other instruments, you should develop an understanding of fluorophore combinations and how they are detected on your specific machine using single fluorophore stained beads to generate compensation matrices. Fluorophores with high spillover have either been omitted or assigned to appropriate markers. As illustrated in Figure 1A, you can evaluate negative spread, relative brightness, and determine more quantitative information, such as fluorophore stain index (SI)^2^^,^^3^ Thus, the more problematic fluorophores can be reserved for markers that are easier to resolve, improving overall panel performance. Understanding of fluorophore interactions is beneficial for both conventional and spectral panel design.

When using a photomultiplier tube (PMT)-based conventional cytometer, as opposed to avalanche photodiodes (APDs), it is important to understand how changes in settings affect the sensitivity for detecting positive events and spreading error. Although there are a number of methods for assessing voltage optimization,^2^ it can be analyzed using eight-peak calibration beads across all detectors while incrementally increasing the voltage of each detector, helping identify an optimal operating window in which all eight bead intensities are clearly distinguishable (Figure 1B). This is particularly important when analyzing large flow cytometry panels as it ensures that positive versus negative populations can be reliably resolved. In some channels, it may be difficult to resolve all eight bead populations. If this occurs, select two resolvable peaks and identify the minimum voltage that achieves maximum separation.

***Note:*** If detector settings are adjusted, use Application settings (or equivalent standardization) to keep them referenced to the daily QC baseline. The daily QC of the instrument indicates the performance of each detector, which is subject to variation. Use of these standardization settings ensures that any differences observed in your data are not due to technical changes.

### Panel design

Successful panel design requires a strong understanding of the chosen cellular markers, including their relative expression levels and whether they are expressed on the same or mutually exclusive cell types. Early in the design process it is important to assess the availability of antibody clones conjugated to less common or novel fluorophores. In addition, having a clear, well-justified gating strategy, using previously optimised antibody clones that are supported by examples in existing literature, will help ensure that positive and negative populations can be optimally resolved. Together, this information guides the assignment of fluorophores to markers and supports the development of a robust, multi-colour panel.

**Figure 2:**
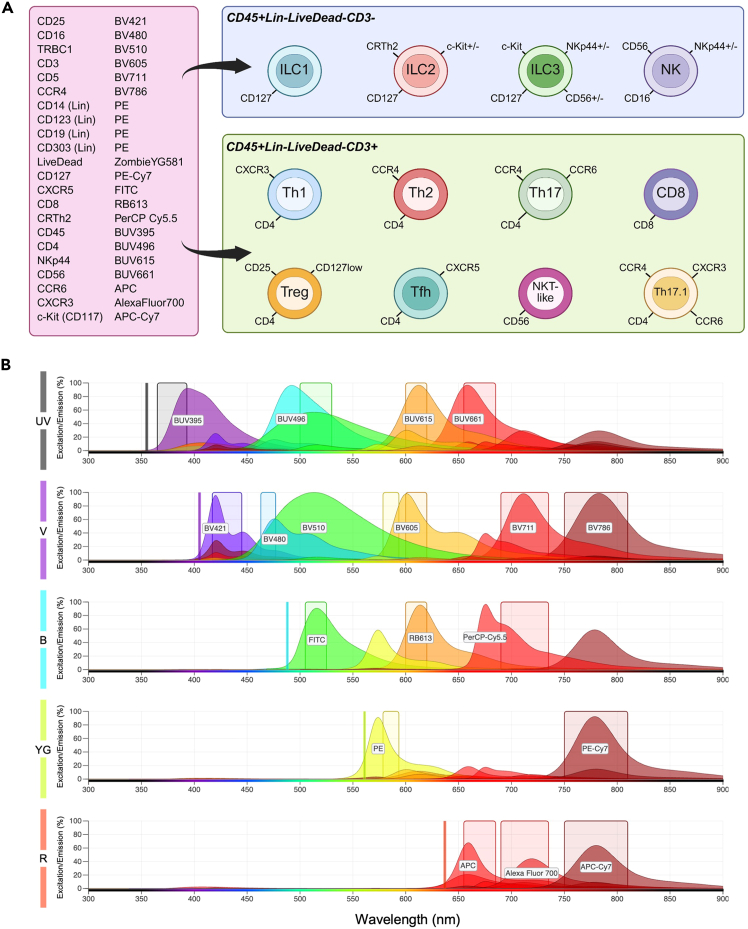
Multi parameter flow cytometer using the BD FACSymphony A3 cytometer allows design of a 19-colour ILC and T cell subset panel (A) Markers and their assigned fluorophores in the final flow cytometry panel as listed in the left panel. Live, CD45 positive, lineage (CD14, CD123, CD19, CD303) negative cells were separated based on their CD3 expression. ILCs and NK cells were defined from the CD3 negative fraction, whereas T cell subtypes were identified from the CD3 positive population. (B) Laser and detector configuration of the BD FACSymphony A3 cytometer with corresponding emission profiles of the fluorophores used in the panel. Fluorochromes were distributed across the ultraviolet (UV), violet (V), blue (B), yellow green (YG), and red (R) lasers. Shaded curves represent relative emission spectra and boxed regions indicate detector bandpass filters assigned to each fluorophore. Illustrations generated using BD® Spectrum Viewer software.

Figure 2A illustrates the markers and corresponding fluorophores included in the optimized panel, together with how their expression patterns define cell populations of interest. In this approach, a single channel is used for both lineage markers and the viability stain due to their exclusion early in our gating hierarchy. However, where possible, these parameters could be detected separately to allow clearer discrimination and flexibility in analysis. As displayed in Figure 2B and listed in Table 1, fluorophores were strategically distributed across the five lasers on the BD FACSymphony A3 to minimize spreading error and support reliable resolution of co-expressed markers.

**Table 1:** BD FACSymphony A3 cell analyzer configuration with corresponding antibody reagents

Laser	Filters	Fluorophore	Relative brightness	Specificity	Clone	Purpose
UV 355nm	379/28	BUV395	2	CD45	HI30	Pan leukocytes
–	515/30	BUV496	2	CD4	SK3	T helper cells
–	610/20	BUV615	3	NKp44	P44-8	ILC3 subsets
–	670/30	BUV661	3	CD56	TULY56	NK Cells
V 405nm	431/28	BV421	4	CD25	BC96	Tregs
–	525/50	BV480	3	CD16 (FcγRIII)	eBioCD16	NK Cells
–	586/15	BV510	2	TRBC1	JOVI.1	T cell clonality
–	610/20	BV605	3	CD5	UCHT2	T cells
–	710/50	BV711	4	CD3	UCHT1	T cells
–	780/60	BV786	3	CCR4	L291H4	T helper 2 and 17 cells
B 488nm	530/30	FITC	2	CXCR5	J252D4	T follicular helper cells
–	610/20	RB613	4	CD8	RPA-T8	Cytotoxic T cells
–	710/50	PerCP-Cy5.5	2	CRTh2	BM16	ILC2s
YG 561nm	586/15	PE	3	CD19	HIB19	B cell exclusion
–	586/15	PE	3	CD14	HCD14	Monocyte exclusion
–	586/15	PE	3	CD123	6h6	Plasmacytoid dendritic cells and eosinophils exclusion
–	586/15	PE	3	CD303	BDCA-2	Myeloid cells and platelet exclusion
–	586/15	Zombie YG581	3	-	-	Viability
–	780/60	PE-Cy7	4	CD127	R34.34	Pan ILCs
R 640nm	670/30	APC	3	CCR6	G034E3	T helper 17 cells
–	710/50	Alexa Fluor 700	1	CXCR3	G025H7	T helper 1 cells
–	780/60	APC-Cy7	1	CD117 (c-Kit)	104D2	ILC3s

Relative brightness is measured as a score out of 4, where 4 is the brightest.

### Innovation

ILC and T cell subsets are critical regulators of immune function, and their dysregulation has been implicated in diseases such as allergy, cancer, and autoimmunity^4^^,^^5^^,^^6^ Our current understanding of these cell populations largely derives from work performed in mouse models,^7^ much of it based on flow cytometric analysis using conventional systems. Alongside this, there is a dual need for the development of robust approaches that enable detailed characterization of their human counterparts and the ability to move between conventional and spectral flow cytometry when designing flow cytometry strategies. Thus, the design of cross-platform, high-parameter panels facilitates the translation of murine findings into human disease contexts, providing a critical tool for modern cytometric analysis.

Here, we present a protocol for high-parameter flow cytometry analysis of human peripheral blood and bone marrow to enable comprehensive and concurrent identification and phenotyping of ILCs and T cell populations. The careful panel design provides a detailed framework to move easily between conventional and spectral cytometry. The panel uses chemokine receptor expression to map T helper and ILC populations, aligning to the new nomenclature literature published by Masopust D. *et al*. (2025)^8^ To address steric hindrance associated with staining small chemokine receptors, we also use a sequential antibody staining strategy that improves epitope accessibility and signal resolution^9^ Additionally, we show how newly available light-loss imaging can be used to identify and potentially then sort immune subsets.

### Institutional permissions

Work with healthy human primary blood cells was performed under approval by the University of York Biology Ethic Committee (DB202111, awarded to Dr. Dave Boucher). Work with human primary blood cells was performed under approval according to REC number 18/YH/0070 (awarded to Prof. Sinisa Savic) for patients with autoinflammatory disorders and REC number 14/WS/0098 (awarded to HMDS, Leeds for surplus material) for patients at diagnosis.

## Key resources table

**Table:** 

REAGENT or RESOURCE	SOURCE	IDENTIFIER
**Antibodies**		

Human anti-CD45 BUV395 (clone HI30, 1:20)	Invitrogen	363-0459-42
Human anti-CD19 PE (clone HIB19, 1:800)	Biolegend	302208
Human anti-CD14 PE (clone HCD14, 1:150)	Biolegend	325605
Human anti-CD123 PE (clone 6h6, 1:100)	Biolegend	306006
Human anti-CD303 PE (clone 201A, 1:100)	Biolegend	354204
Human anti-CD3 BV605 (clone UCHT1, 1:100)	Biolegend	300459
Human anti-CD4 BUV496 (clone SK3, 1:500)	Invitrogen	3640047-42
Human anti-CD8 RB613 (clone RPA-T8, 1:20)	BD	571089
Human anti-CD56 BUV661 (clone TULY56, 1:80)	Invitrogen	3760566-41
Human anti-CD16 BV480 (clone eBioCD16, 1:80)	Invitrogen	414-0168-41
Human anti-NKp44 BUV615 (clone P44-8, 1:160)	BD	752353
Human anti-CRTh2 PerCP-Cy5.5 (clone BM16, 1:50)	Biolegend	350115
Human anti-CD117 APC-Cy7 (clone 104D2, 1:50)	Biolegend	313227
Human anti-CD127 PE-Cy7 (clone R34.34, 1:60)	Invitrogen	25-1271-42
Human anti-TRBC1 Biotin (clone JOVI.1, 1:80)	Biolegend	383512
Human anti-CD5 BV711 (clone UCHT2, 1:300)	BD	563170
Zombie YG581 Live Dead Stain (1:2000)	Biolegend	423123
Human anti-CXCR3 Alexa Fluor 700 (clone G025H7, 1:20)	Biolegend	353742
Human anti-CXCR5 FITC (clone J252D4, 1:20)	Biolegend	356914
Human anti-CD25 BV421 (clone BC96, 1:20)	Biolegend	302630
Human anti-CCR6 APC (clone G034E3, 1:20)	Biolegend	353416
Human anti-CCR4 BV786 (clone L291H4, 1:20)	Biolegend	359448
Streptavidin BV510 (1:200)	Biolegend	405233
Human anti-CRTh2 FITC (clone BM16, 1:50)	Biolegend	350107
Human anti-NKp44 APC (clone P44-8, 1:160)	Biolegend	325109
Human anti-CD117 BV421 (clone 104D2, 1:50)	Biolegend	313215
Human anti-CD8 BUV805 (clone RPA-T8, 1:80)	Invitrogen	368-0088-41

**Chemicals, peptides and recombinant proteins**		

BD Horizon Brilliant Stain Buffer	BD	563794
UltraComp eBeads™ Compensation Beads	Invitrogen	01-2222-41
Dulbecco’s Phosphate Buffered Saline	Sigma-Aldrich	D8537-500ML
Foetal bovine serum	Gibco	10270106
Tris	Invitrogen	15504-020
Ammonium Chloride NH_4_Cl	Sigma-Aldrich	A9434

**Biological samples**		

Human peripheral blood and bone marrow samples	Hematological Malignancy Diagnostic Service, St James’ Hospital, Leeds	N/A
Healthy adult peripheral blood	University of York Tissue Bank	N/A
Human peripheral blood from patients with autoinflammatory disease	Institute of Rheumatic and Musculoskeletal Medicine, Leeds	N/A

**Other**		

Pre-Separation Filter 20 μM	Miltenyi Biotec	130-101-812
FACSymphony A3	BD	N/A
FACSDiscover S8	BD	N/A

## Materials and equipment

**Table:** Staining buffer

Reagent	Final concentration	Amount
Phosphate Buffered Saline (PBS) 1×	N/A	500 mL
Foetal Bovine Serum (FBS)	2%	10 mL

***Note:*** Mix thoroughly and store at 4^o^C. Prepare the day before or on the day of the experiment.

**Table:** RBC lysis buffer

Reagent	Final concentration	Amount
TRIS	N/A	1.03 g
Ammonium chloride (NH_4_Cl)	N/A	3.74 g
Autoclaved Milli-Q H_2_O	N/A	500 mL

***Note:*** Adjust the pH to 7.2 and filter sterilize before use. Store at 19^o^C for up to 6 months.

## Step-by-step method details

### Antibody titrations

**Timing: 4 h (per antibody titration)**

Here, we describe steps for titrating antibodies to identify their optimal concentration.

***Note:*** Antibody titrations optimize separation of positive and negative populations via SI calculations^2^^,^^3^ They reduce spread in the negative population, lower antibody use, and prevent oversaturation and non-specific binding. Antibodies should be titrated individually on the relevant tissue and in an order aligned with the gating strategy. If antibodies used in earlier gates are not optimally titrated, it can compromise the gating of downstream populations, leading to inaccurate results.

1. Prepare the sample exactly as for the full experiment, using the same tissue type and processing methods (see sample preparation section).
  a. Prepare a minimum of eight sample wells, ensuring that each well contains as close to the intended cell number as possible.

***Note:*** Any deviation in cell density, tissue source, or preparation protocol in the final experiment may alter antibody binding, background signal, and the overall quality of the data.

**CRITICAL:** When titrating antibodies, follow the order of your predicted gating strategy, starting with the first antibody in your hierarchy. Once the optimal concentration of the first antibody has been determined, use it at this concentration in the staining buffer when titrating your second antibody. Continue this sequential process, cumulatively adding each optimized antibody to subsequent titrations so that every antibody is titrated in the context of those that precede it.

2. Prepare a two-fold serial dilution of the antibody being titrated across a minimum of six dilution steps.
  a. If this is the first antibody being titrated, dilute the antibody in staining buffer only.
  b. Dilute any subsequent antibodies in staining buffer that contains the previously titrated antibodies.
  c. Start the serial dilution at approximately twice the manufacturer’s recommended concentration.
3. Stain equivalent cell numbers with each antibody dilution for 40 min, in the dark at 4^o^C.
  a. Include an unstained control where no antibodies are added.
  b. From the second antibody titration onwards, include a fluorescence minus one (FMO) control for the antibody being titrated.

***Note:*** An FMO is where the antibody of interest is omitted from the sample but antibodies against other cellular markers are still included. An FMO enables background and spillover fluorescence to be accounted for, and so gating boundaries can be accurately defined.

4. Wash off the antibody stain.
  a. Add 200 μL of staining buffer.
  b. Centrifuge the sample (300 *× g*, 5 min).
  c. Discard the supernatant.
  d. Resuspend each sample in 200 μL of staining buffer.
5. Analyze each sample on the cytometer.
6. Calculate SI and plot values to identify the optimal antibody concentration.^2^^,^^3^
  a. Take away the median fluorescence intensity (MFI) of the negative population from the MFI of the positive population, then divide this difference by twice the standard deviation of the negative population.
    $$\text{Stain}\;\text{Index}=\frac{{\text{MFI}}_{\text{pos}}-{\text{MFI}}_{\text{neg}}}{{2\sigma }_{\text{neg}}}$$
    MFI = Median Fluorescence Intensity.
    σ = Standard Deviation.
  b. Plot SI against antibody concentration.
    ***Note:*** The optimal concentration is the point at which the SI graph plateaus; this is where the antibody concentration is at saturation^2^^,^^3^
  c. Visually inspect the negative spread and separation between positive and negative populations.
    i. Concatenate the flow cytometry standard (FCS) files.
    ii. Generate dot plots of time versus the fluorophore being investigated.
    iii. Identify the concentration that generates the greatest separation between positive and negative populations with the least negative spread in the negative population.
  d. See problem 4 if an optimal concentration cannot be obtained.
7. To titrate the Zombie YG581 viability dye, repeat this protocol, making the following changes.
  a. Kill 50% of each cell sample by heating to 80^o^C for 5 min.
  b. Perform all dilutions in PBS only (without serum).
    **CRITICAL:** 2% FBS impacts Zombie YG581 staining so the cell sample must be washed with PBS only, not staining buffer, prior to staining with Zombie YG581. Zombie YG581 must also be diluted in PBS for this same reason. If your cells require 2% FBS, you can titrate the Zombie YG581 in the presence of 2% FBS.
  c. Before adding the diluted Zombie YG581 to the sample, wash off the FBS present in the sample.
    i. Resuspend each pellet in 200 μL of PBS only (without serum).
    ii. Centrifuge the sample (300 *x*g, 5 min).
    iii. Discard the supernatant.
  d. Add 50 μL of Zombie YG581 and incubate for 15 min on ice, in the dark.
  e. Wash off the Zombie YG581.
    i. Add 200 μL of staining buffer.
    ii. Centrifuge the sample (300 *× g*, 5 min).
    iii. Discard the supernatant.
  f. Resuspend the pellet in 200 μL of staining buffer.
  g. Analyze sample on the cytometer.

### Preparation of single-stain controls

**Timing: 1 to 2 h**

This section outlines how to generate single-stain controls to use in compensation or spectral unmixing.

***Note:*** We recommend using antibody capture beads (see key resources table) for generating single-stain controls. These beads consist of a non-staining, negative reference population and an antibody-binding population, providing a brighter fluorescent signal than stained cells. This ensures that any spectral spillover can be precisely measured and corrected. You should check the species compatibility of your beads against your antibodies.

***Note:*** We advise the use of the auto-compensation function, which is available on most cytometer softwares. Due to the simultaneous detection of PE and Zombie YG581, an independent control must be generated on cells, instead of compensation beads, for this channel, as the cell viability dye will not be recognised by beads.

8. Generate single-stained controls using beads.
  a. Vortex the compensation beads thoroughly for 30 s.
  b. Add 1 μL of each antibody to one drop of beads.
  c. Incubate for 30 min on ice and in the dark.
    **CRITICAL:** For the streptavidin-conjugated antibody, use a different antibody that is directly conjugated to BV510. Streptavidin-conjugated reagents do not bind to beads in the same reliable way as directly-conjugated antibodies, meaning the staining with beads may not be bright or consistent enough.
    ***Note:*** For conventional flow cytometry, an unstained bead control was not required as a negative portion within each single stain-control was used.
  d. Wash off any excess antibody.
    i. Add 200 μL of staining buffer.
    ii. Centrifuge the samples (300 *× g*, 5 min).
    iii. Discard the supernatant.
  e. Resuspend in 200 μL of staining buffer.
    ***Note:*** Accounting for background signal is particularly important for correct spectral unmixing, therefore, we advise using a separate unstained bead control when analyzing samples spectrally.
    ***Note:*** When using a conventional cytometer, PE and Zombie YG581 are detected by the same channel, therefore, a control sample that contains both fluorophores must be used for compensation.
9. Prepare a Zombie YG581 and PE combined control sample.
  a. Take ∼200,000 RBC lysed cells and kill 50% by heating to 80^o^C for 5 min.
  b. Incubate the dead cells on ice for 5 min.
  c. Combine the dead cells with the remaining live cells.
  d. Centrifuge the samples (300 *× g*, 5 min).
  e. Discard the supernatant.
  f. Resuspend the pellet in a 50 μL antibody cocktail that contains only PE Lineage antibodies, made up using the staining buffer.
    ***Note:*** See Table 2 for PE lineage antibody dilutions.
  g. Incubate the samples for 40 min on ice and in the dark.
  h. Wash off the antibody cocktail.
    i. Add 200 μL of PBS only (without serum).
    ii. Centrifuge the samples (300 *× g*, 5 min).
    iii. Discard the supernatant.
  i. Resuspend the pellet in 50 μL of diluted Zombie YG581 (1 in 2000, diluted in PBS only, without serum).
  j. Incubate on ice, in the dark, for 15 min.
  k. Wash off the Zombie YG581.
    i. Add 200 μL of staining buffer.
    ii. Centrifuge the samples (300 × *g*, 5 min).
    iii. Discard the supernatant.
  l. Resuspend the pellet in 200 μL staining buffer.
  m. Analyze the sample on the cytometer.
    ***Note:*** If running this panel on a spectral cytometer, an independent Live Dead control must be generated because each fluorochrome needs to be evaluated uniquely.
10. Generate an independent Live Dead control.
  a. Take ∼200,000 RBC lysed cells and kill 50% by heating to 80°C for 5 min.
  b. Incubate the dead cells on ice for 5 min.
  c. Combine the dead cells with the remaining live cells.
  d. Before staining with Zombie YG581, wash off the FBS present in the sample.
    i. Add 200 μL of PBS only (without serum).
    ii. Centrifuge the samples (300 × *g*, 5 min).
    iii. Discard the supernatant.
  e. Resuspend the pellet in 50 μL of Zombie YG581 (diluted 1 in 2000 in PBS only).
  f. Incubate on ice, in the dark, for 15 min.
  g. Wash off the Zombie YG581.
    i. Add 200 μL of staining buffer.
    ii. Centrifuge the sample (300 × *g*, 5 min).
    iii. Discard the supernatant.
  h. Resuspend the pellet in 200 μL of staining buffer.
11. Run single-stained controls on the cytometer.
  **CRITICAL:** Standardisation settings must be applied to your experiment before running compensation controls. If these are applied afterwards, compensation controls must be run a second time with the adjusted cytometer settings.
  a. If using application settings or equivalent standardisation, apply these to your experiment before acquiring compensation controls.
  b. Set up compensation controls (conventional cytometer) or single-stained samples (spectral cytometer) in your cytometer software.
  c. Record a minimum of 5,000 bead events per single-stained compensation control. For viability (live/dead and PE/Zombie YG581) controls, record up to 50,000 events.
  d. Perform auto-compensation on a conventional cytometer.
    i. Gate on both the positive and negative populations for each control.
    ii. Apply the auto-compensation function in the software to calculate the compensation matrix.
  e. Perform spectral unmixing on a spectral cytometer.
    i. Run the unstained beads and unstained cells.
    ii. Gate on the positive population for each stained control.
    iii. Follow the software’s instructions to perform spectral unmixing.

**Table 2:** Antibody dilutions and sample contents

Conjugate	Specificity	Dilution	Full stain	+CD127 control	-CD127 control	CD5 FMO	CD16 FMO	T panel FMO	Oher
BUV395	CD45	1:20	✔	✔	✔	✔	✔	✔	✔
BUV496	CD4	1:500	✔	✔	✔	✔	✔	✔	✔
BUV615	NKp44	1:160	✔	X	X	X	X	✔	X
BUV661	CD56	1:80	✔	X	X	X	✔	✔	X
BV421	CD25	1:20	✔^a^	X	X	X	X	X	X
BV480	CD16 (FcγRIII)	1:80	✔	✔	✔	✔	X	✔	X
Biotin	TRBC1	1:80	✔	X	X	X	X	✔	X
BV605	CD5	1:300	✔	✔	✔	X	✔	✔	✔
BV711	CD3	1:100	✔	✔	✔	✔	✔	✔	✔
BV786	CCR4	1:20	✔^a^	X	X	X	X	X	b
FITC	CXCR5	1:20	✔^a^	X	X	X	X	X	b
RB613	CD8	1:20	✔	X	X	X	X	✔	X
PerCP-Cy5.5	CRTh2	1:50	✔	X	X	X	X	X	X
PE	CD19	1:800	✔	✔	✔	✔	✔	✔	✔
PE	CD14	1:150	✔	✔	✔	✔	✔	✔	✔
PE	CD123	1:100	✔	✔	✔	✔	✔	✔	✔
PE	CD303	1:100	✔	✔	✔	✔	✔	✔	✔
Zombie YG581	-	1:2000	✔^a^	✔^a^	✔^a^	✔^a^	✔^a^	✔^a^	✔^a^
PE-Cy7	CD127	1:60	✔	✔	X	X	X	✔	X
APC	CCR6	1:20	✔^a^	X	X	X	X	X	b
Alexa Fluor 700	CXCR3	1:20	✔^a^	X	X	X	X	X	b
APC-Cy7	CD117 (c-Kit)	1:50	✔	X	X	X	X	✔	X
Strep-BV510	Biotin	1:80	✔^a^	✔^a^	X	X	X	✔^a^	X

a indicates that these antibodies should not be added to the antibody cocktail and instead added independently. See protocol steps for more detail.

b These should be added separately to create controls where only one of these antibodies is added. For example, a CXCR3 only control will not contain CCR4, CCR6, or CXCR5.

### Sample preparation

**Timing: 1 h**

The section below describes how to prepare the bone marrow or peripheral blood tissue prior to flow cytometry staining.

12. For every 1 mL of peripheral blood or bone marrow, dilute in 10 mL of RBC lysis buffer.
  a. Incubate for 10 min at 37^o^C.
13. Wash the sample.
  a. Centrifuge the sample (300 *×g*, 5 min).
  b. Remove the supernatant.
  c. Resuspend the pellet in 200 μL of staining buffer.
  d. If cell aggregates are present, filter the cell suspension through a 20 μM filter.
14. Repeat step 13 once more.
15. Check cell counts and viability.
  a. Resuspend the pellet in a known volume.
  b. Count how many cells are present
  c. Check cell viability.
16. Aliquot the sample for fully stained and control samples.

### Cell staining for human peripheral blood and bone marrow samples

**Timing: ∼4 h**

In this section, we detail how to stain your prepared tissue and perform flow cytometry analysis.

**Figure 3:**
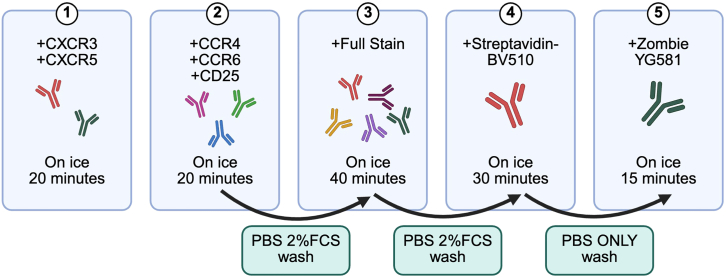
Schematic for sequential staining protocol Antibodies for chemokine receptors are added first, followed by the remaining antibody cocktail, streptavidin-BV510, and then the Zombie YG581 viability dye.

***Note:*** Staining of some chemokine receptors, such as CXCR3 and CXCR5, can be difficult due to the small size of the proteins and steric hindrance. Therefore, this protocol makes use of sequential staining (Figure 3), whereby these antibodies are added independently, prior to the fully stained cocktail.^9^

***Note:*** In addition to the fully stained antibody cocktail, there are additional controls that are used to set gates for lowly expressed markers, and these are described in Table 2.

17. Centrifuge the sample (300 *× g*, 5 min) and resuspend each pellet in 50 μL of staining buffer.
18. Directly add the CXCR3 and CXCR5 antibodies.
  a. Incubate on ice, in the dark, for 20 min.
19. Directly add the CD25, CCR6, and CCR4 antibodies.
  a. Incubate on ice, in the dark, for 20 min.
20. Wash off the CXCR3, CXCR5, CD25, CCR6, and CCR4 antibodies.
  a. Add 200 μL of staining buffer.
  b. Centrifuge the samples (300 *× g*, 5 min).
  c. Discard the supernatant.
21. Resuspend the pellet in the relevant antibody cocktail.
  a. Incubate on ice, in the dark, for 40 min.

***Note:*** See Table 2 for antibody cocktail details.

***Note:*** We recommend making the antibody cocktails on the day of the experiment, using 7% BD Horizon Brilliant Stain Buffer. This will prevent interactions between antibodies, particularly the BV and BUV dyes, reducing non-specific binding.

***Note:*** Prepare antibody cocktails with at least 10% excess volume to ensure sufficient reagent is available for staining samples.

***Note:*** To prolong antibody quality, keep all antibodies at 4^o^C or on ice during cocktail preparation, protected from light, and with caps tightly sealed to minimize air exposure and prevent oxidation of tandem dyes.

22. Wash off the antibody mix.
  a. Add 200 μL of staining buffer.
  b. Centrifuge the samples (300 *× g*, 5 min).
  c. Discard the supernatant.
23. Resuspend the pellet in 50 μL of Streptavidin BV510.
  a. Incubate on ice, in the dark, for 30 min.
24. Wash off the Streptavidin BV510 and FCS present in the sample.
  a. Add 200 μL of PBS only (without serum).
  b. Centrifuge the samples (300 *× g*, 5 min).
  c. Discard the supernatant.
25. Resuspend the pellet in 50 μL of Zombie YG581 (1 in 2000, diluted in PBS only, without serum).
  a. Incubate in the dark at 4^o^C for 15 min.
26. Wash off the Zombie YG581.
  a. Add 200 μL of staining buffer.
  b. Centrifuge the samples (300 *× g*, 5 min).
  c. Discard the supernatant.
27. Resuspend the pellet in 200 μL of staining buffer.
28. Analyze samples on the cytometer.
29. Analyze the data.
  a. Use appropriate software, such as FlowJo 10.10.0 (FlowJo Software, BD).
  b. Generate scatter plots to identify different populations of cells and histograms to further investigate marker expression.

## Expected outcomes

### Final gating strategy

**Figure 4:**
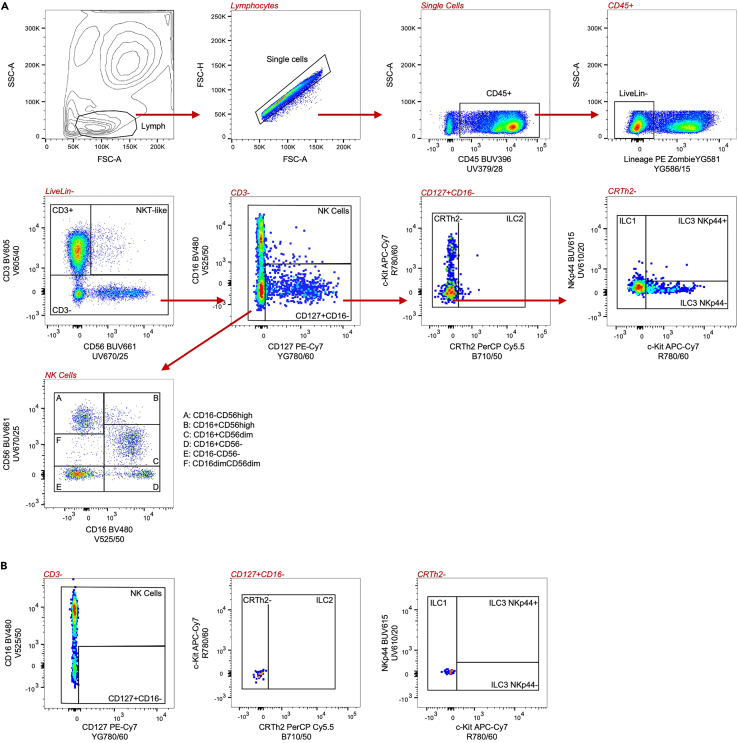
Gating strategy for identifying ILC subtypes and NK cells (A) Gating strategy used for identifying NK cells and ILC subtypes. Red italics indicate parent gate. (B) FMO controls used for placing final ILC gates. Analysis performed using BD FlowJo 10.10.0 software.

**Figure 5:**
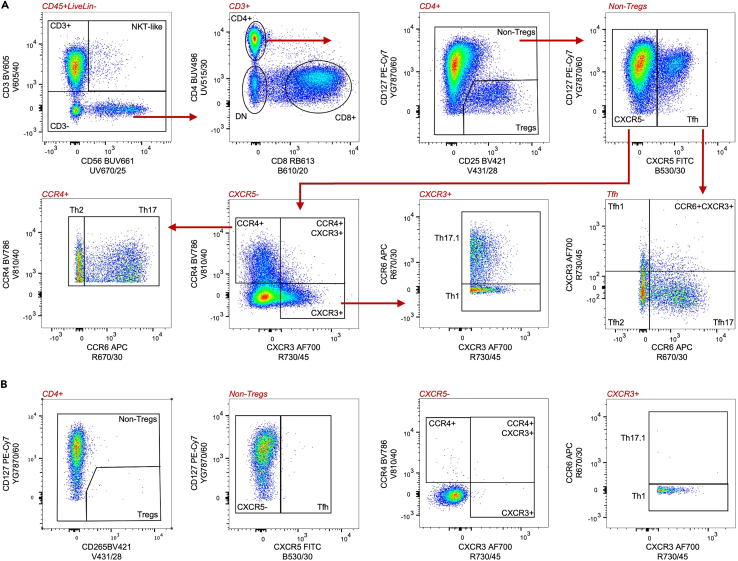
Gating strategy for identifying different T cell subsets (A) Gating strategy used for identifying various T cell populations. Red italics indicate parent gate. (B) FMO controls used for placing gates. Analysis performed using BD FlowJo 10.10.0 software.

As circulating ILCs are present in very low frequencies in healthy individuals, the example gating strategy deriving from the protocol is taken from a patient with autoinflammatory disease to ensure adequate visualization of these populations. Healthy donor samples were nevertheless included during panel optimization and validation. After initial gating on lymphocytes and singlets, CD45^+^ cells were selected. Lineage^+^ and dead cells were then excluded. At the CD3 versus CD56 gating step, the analysis diverges for ILCs and NK cells (Figure 4) and a concurrent analysis of T cell subsets (Figure 5). Pan-ILCs are identified as CD45^+^Lin^-^CD3^-^CD16^-^CD127^+^, with CD16 used as the NK cell exclusion marker in preference to CD56, as CD56 expression can also occur on subsets of ILCs^4^ Furthermore, multiple ILC subsets are defined based on their CRTh2 and c-Kit expression, including ILC1, ILC2, and finally ILC3 populations, distinguishable by the NKp44 marker. In parallel, six NK cell populations were resolved based on their differential CD56 and CD16 expression. As CD127 is critical for ILC identification, an FMO control is routinely used to support accurate gate placement. An additional FMO lacking CRTh2, c-Kit, and NKp44 was included, as these markers are primarily expressed on small populations of cells.

Figure 5 demonstrates how this high-parameter flow cytometry panel can identify up to eleven distinct T cell subsets. The strategy applied here primarily harnesses chemokine receptor expression, which not only enables detailed T cell phenotyping but also allows parallel assessment of these receptors on ILC populations, which is less well understood. T cell analysis could be extended further, as the panel includes additional markers with important biological relevance, including CD127, TRBC1, CD5, and CRTh2. For example, CD8^+^ T cells can be further interrogated by examining CD127 and CXCR3 expression, in line with the novel T cell nomenclature^6^

Overall, two manual gating strategies are presented that together identify more than 20 immune cell populations. However, the breadth of markers included in this panel provides scope for deeper phenotypic interrogation, enabling further subdivision of these populations and potential insights into the function of these cell populations.

### Translating a conventional panel to spectral

**Figure 6:**
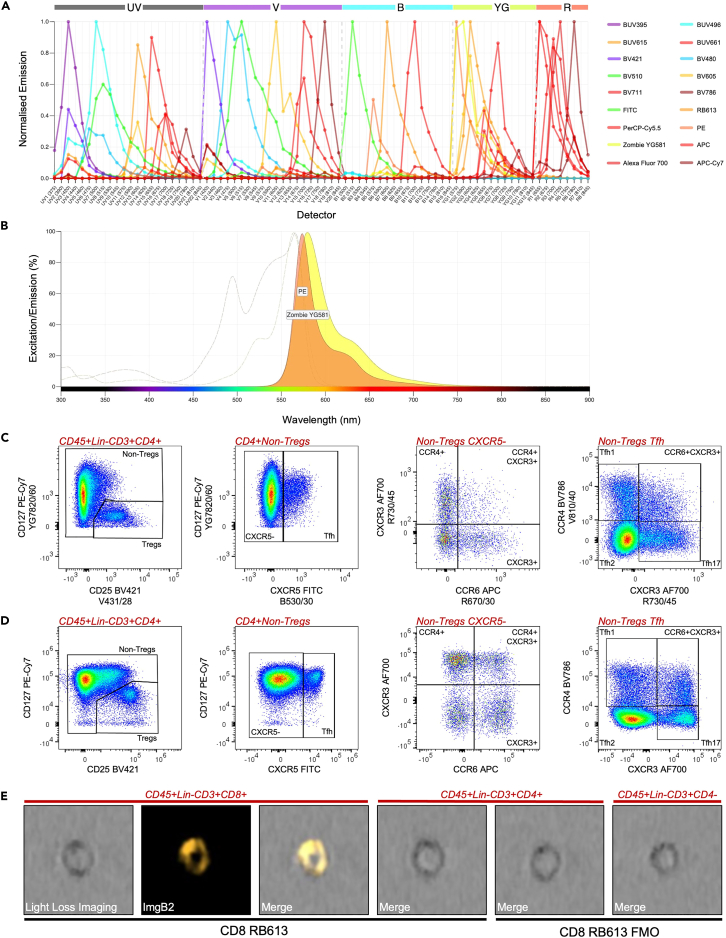
Spectral analysis of the 19-colour ILC and T cell panel provides greater resolution than conventional analysis (A) Emission profiles for the 19-colour panel created using the BD spectrum viewer. This generated a spectral complexity score of 7.4. (B) Excitation and emission profiles for PE and Zombie YG581 were created using BD Spectrum viewer. (C) Peripheral blood from healthy human donors were analyzed conventionally on the BD FACSymphony A3 or (D) spectrally on the BD FACSDiscover S8 cytometer. Plots show a selection of gates employed in T cell identification and are representative of six donors. Analysis performed using BD FlowJo 10.10.0 software. (E) Representative images captured on the BD FACSDiscover S8. Panel shows light loss imaging, RB613 emission captured using the 488 nm ImgB2 channel with 600/60 bandpass filter, and merged images of both modalities. Cells were stained either with or without (FMO) CD8 RB613, as indicated below each image. The gate that each cell was located is shown in red italics above the corresponding image.

Due to the in-depth experimental design and antibody titrations, this panel is readily transferable to spectral cytometry because fluorochromes are resolved based on their full emission profile, rather than peak emission alone. Figure 6A shows the spectral emission plot for the fluorophores in this panel, which yields a spectral complexity score of 7.4. This score is a metric used to predict the difficulty in spectrally unmixing a given panel of fluorochromes, based on how similar their emission spectra are. In contrast, a panel optimized for one conventional cytometer cannot always be directly transferred to another, as differences in filter configurations, detector sensitivity, and optical layouts alter the data generated. In addition, transferring a panel optimized for a spectral instrument is considerably more challenging, as conventional systems may lack the necessary lasers and filters, and are unable to resolve closely overlapping fluorophores.

Notably, PE and Zombie YG581 can be resolved independently during spectral analysis. As illustrated in Figure 6B, these fluorochromes exhibit very similar emissions. However, their distinct spectral signatures allow them to be spectrally unmixed accurately, which allows retention of the original panel design. Equally, if the panel was designed only for spectral analysis an alternative viability stain could be used.

Astakhova et al., discuss, in-depth, the general differences between conventional and spectral flow cytometry,^1^ whereas Table 3 highlights the key differences between the FACSymphony A3 conventional cytometer and the FACSDiscover S8 spectral cytometer. When comparing conventional and spectral acquisition, spectral analysis provides improved population resolution (Figures 6C and 6D). This improvement could be due to many factors, including the use of APD rather than PMTs or analysis of the whole vs. partial spectra. This enhances the ability to distinguish dim or closely related populations, which is particularly important in high parameter immunophenotyping, where marker co-expression is common.

**Table 3:** Comparison between BD FACSymphony A3 and FACSDiscover S8 cytometers

	FACSymphony A3	FACSDiscover S8
Cytometer type	Conventional	Spectral
Sorting capability	None, analyzer only	6-way sorting into tubes or plate
Lasers	5	5
Detector number	31	78
Detector type	Photomultiplier tubes	Avalanche photodiode
Imaging capability	No	Yes
Autofluorescence removal	No	Yes

The BD FACSDiscover S8 also incorporates light-loss imaging, which generates cell images based on reductions in transmitted light. These images improve gating precision by allowing the removal of debris and doublets. Moreover, quantitative metadata is generated for each image, including morphological and spatial features such as centre of mass, eccentricity, and diffusivity, enabling detailed characterisation of individual cells. Fluorescence imaging can also be performed using the 488 nm laser. Figure 6E shows imaging of CD8 RB613 on cells compared with the appropriate controls. Moreover, these morphological insights are generated in real-time, meaning that they can be employed in sorting strategies. For example, cells exhibiting localized fluorescence within the nucleus or at the membrane can be identified and selected^10^ When using the BD FACSDiscover S8 it is important that the same staining buffer is used for all samples, including single-stained controls, across all experiments, to ensure accurate unmixing.

## Limitations

One limitation of this study is that PE and Zombie YG581 are detected by the same channel when analyzed using conventional flow cytometry, presenting challenges for compensation. However, PE- and Zombie YG581-positive events show minimal spillover into other detectors and are excluded at an early stage of the gating strategy. Therefore, the impact of this issue on downstream analysis is limited. This panel has been optimized specifically for human peripheral blood and bone marrow samples. As a result, additional optimization may be required before it can be reliably applied to other human tissue types. The antibodies included in this panel are specific to human antigens and so mouse-specific equivalents would need to undergo a full optimization process prior to the use in murine samples. Furthermore, marker selection would need to be adapted for use in mice, as certain subset-defining markers differ between species. For example, murine ILC2s are characterized by ST2 expression, whereas human ILC2s are identified by CRTh2.^4^

## Troubleshooting

### Problem 1

Despite careful panel design to minimize spillover, panels of this size inevitably include fluorophores with overlapping emission spectra (step, ‘before you begin’, panel design), therefore spillover cannot be completely eliminated. Spillover increases background signal in affected channels, broadens signal distribution, and can reduce resolution between the positive and negative populations. This loss of resolution is particularly problematic for dim markers, where spillover-induced spread can obscure biologically meaningful differences.

### Potential solution

The continuous development of novel fluorophores provides opportunities to reduce spillover-associated spread in complex panels. Recently released dyes, such as the BD Horizon Real Blue, Yellow, Red, and Violet series, are designed with improved spectral properties, including narrower emission profiles and reduced spread into neighboring detectors, despite maintaining high brightness. Remaining up to date with available fluorophore options can therefore be highly valuable when designing and optimizing panels.

**Figure 7:**
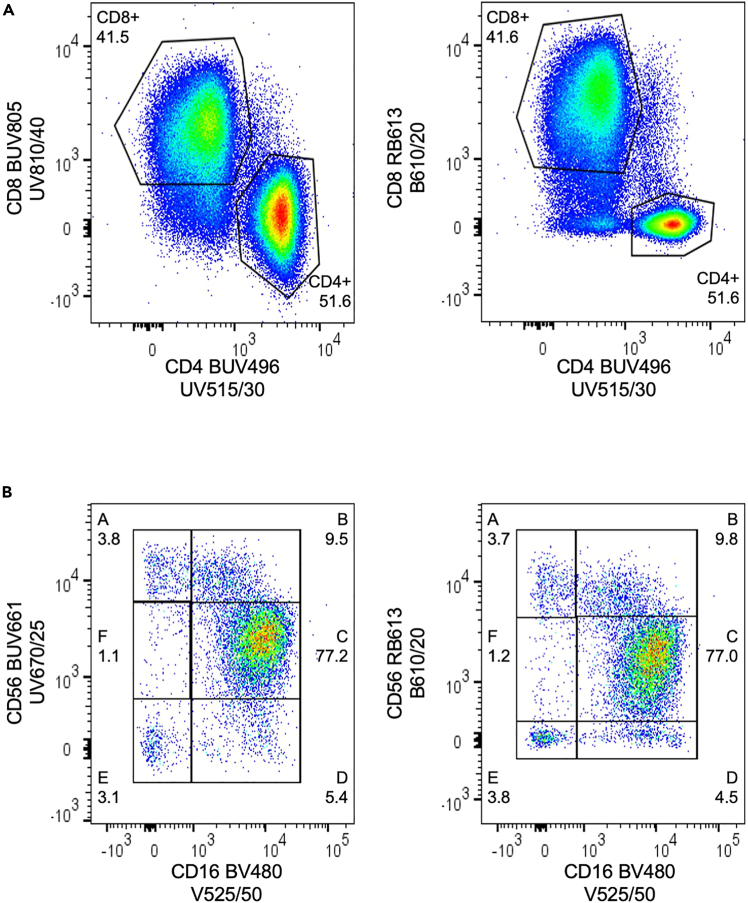
Substituting BUV805 for RB613 reduces spillover and improves population resolution (A) Dot plots showing CD4 BUV496 versus CD8 conjugated to BUV805 or RB613. (B) Dot plots showing CD16 BV480 versus CD56 conjugated to BUV661 or RB613. Analysis performed using BD FlowJo 10.10.0 software.

For example, here BUV805 and BUV661 were identified as fluorophores contributing most significantly to compensation complexity. As shown in Figure 7, substituting these fluorophores for RB613 was investigated. Replacing BUV805 with RB613 reduced the spread observed in the BUV496-positive population, improving separation and overall population definition. Compensation matrices (data not shown) also indicated less extreme spillover values following this substitution.

### Problem 2

The ILC subsets exist in relatively low frequencies, which introduces uncertainty when distinguishing true positive events from background signals (step 21 and 29 and Table 2). Small amounts of spillover or non-specific binding can create events that appear phenotypically positive, but do not represent genuine cells. This is particularly relevant in high-parameter panels where cumulative spread from multiple fluorophores can influence population resolution.

### Potential solution

**Figure 8:**
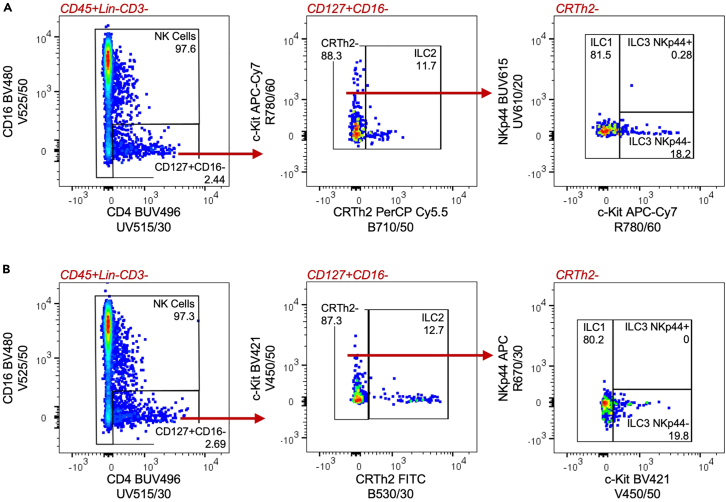
Validation of challenging markers by varying fluorophore conjugates (A) Gating strategy using the established antibody panel for identifying ILC subtypes. (B) Equivalent gating strategy using alternative antibody conjugates with improved spectral properties. NK cell and CD127+CD16-gating remained consistent across the two panels. Similar levels of ILC2s were identified, despite differences in brightness. The proportion of ILC1s and ILC3s was comparable across both groups of antibodies. Plots are representative of 3 biological repeats. Analysis performed using BD FlowJo 10.10.0 software.

To ensure precise gating of rare populations, we suggest FMO controls to provide the necessary boundary definitions (Figure 4; Table 2). Antibodies of concern can often be validated using alternative conjugates with more optimal spectral properties, such as BV421, FITC, and APC. This was feasible in this panel because these fluorophores were assigned to markers reserved for T cell analysis and therefore not required for ILC gating. As evident in Figure 8, the percentage of cells present in each gate was comparable between the same antibody conjugated to different fluorophores, thereby increasing confidence in the suitability of the selected antibodies.

### Problem 3

**Figure 9:**
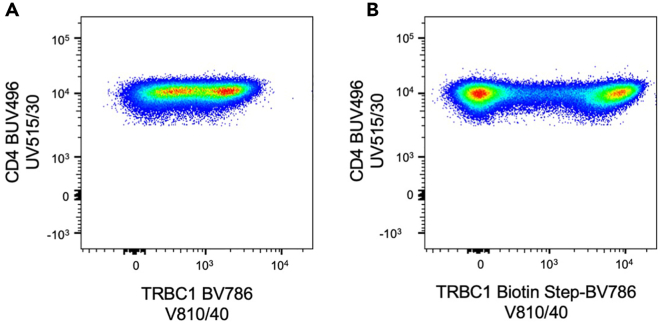
Biotin conjugates can improve sub-optimal staining Human peripheral blood was stained with either (A) TRBC1 directly conjugated to BV786 or (B) TRBC1 conjugated to biotin followed by incubation with streptavidin-BV786. Dot plots showing TRBC1 expression on CD45^+^Lin^-^CD3^+^CD4^+^ cells. Analysis performed using BD FlowJo 10.10.0 software.

TRBC1 was initially conjugated to BV786 (step 21), however, this pairing failed to provide adequate separation between positive and negative populations, as evidenced in Figure 9A. Since TRBC1 is used to identify T cell clonal populations, clear and reliable discrimination between these populations is required.

### Potential solution

To improve TRBC1 staining, TRBC1 conjugated to biotin was investigated, followed by incubation with streptavidin-BV786. As shown in Figure 9B, this approach did yield improved TRBC1 staining. The enhanced separation is likely attributable to increased signal amplification, as the streptavidin-biotin interaction allows multiple fluorochrome-conjugated streptavidin molecules to bind a single biotinylated antibody. Given these results, the biotin-streptavidin strategy was retained when TRBC1 was later paired with BV510.

### Problem 4

An appropriate antibody concentration could not be determined from the titration data (step 6), as SI calculations failed to reveal a clear plateau and visual inspection showed no notable differences between antibody concentrations.

### Potential solution

This often occurs when the cells expressing the target antigen are present in low frequencies. For example, NKp44, which in this protocol is used to identify ILC3s, is expressed on a relatively rare cell population. To address this, either the total number of cells analyzed can be increased, or the titration can be assessed on alternative cell types that are known to have greater expression of the marker.

### Problem 5

There is a low number of events captured during the flow cytometry acquisition (step 28).

### Potential solution

A possible explanation is a low cell yield, which may result from issues during the centrifugation and washing steps (step 4, 7-10, 13, 17, 20, 22, 24, or 26). To mitigate this, ensure that a visible pellet is formed at the end of each centrifugation.

### Problem 6

No staining was observed on single-stained bead controls (step 11).

### Potential solution

Ensure that the antibody capture beads are compatible with the species of the primary antibodies being used, as incompatibility will prevent the antibody from binding to the surface of the beads. An alternative suggestion is that a directly conjugated antibody was not used in place of the streptavidin conjugated antibody when generating this single-stain control.

### Problem 7

Compensation or unmixing errors occur (step 11).

### Potential solution

Check that the single-stained controls are sufficiently bright with good separation between the positive and negative populations, and remake any that do not meet this criteria. Verify that the gates are correctly placed to capture positive and negative events, and confirm that the cytometer machine settings are identical between single-stained controls and the experimental samples, as any discrepancies will introduce errors. Finally, be aware that tandem dyes, such as PE-Cy7, are susceptible to degradation and slight differences between antibody species (for example batch or clone), which can alter their emission profiles. Ensure these reagents have been stored correctly, are within their expiry date, and you are using the same antibody as in your mixed sample. Additionally, it is sometimes possible that the spectral profile of fluorochrome-stained beads may not be identical to that of stained cells. In this case a cell-based single-stain control should be used for accurate unmixing.

## Resource availability

### Lead contact

Further information and requests for resources and reagents should be directed to and will be fulfilled by the lead contact, Dr. Jillian L. Barlow (jillian.barlow@york.ac.uk).

### Technical contact

Technical questions on executing this protocol should be directed to and will be answered by the technical contact, Ms. Jadie Acklam (ja1109@york.ac.uk).

### Materials availability

This study did not generate new or unique reagents.

### Data and code availability

The dataset/code supporting the current study have not been deposited in a public repository as they are not yet part of a published article, but are available from the technical or lead contact on request.

## Acknowledgments

We are grateful to the patients and healthy volunteers who donated their samples for this research and to the Hematology Malignancy Diagnostic Service (HMDS) for providing access to samples and for the use of their BD FACSymphony A3 cytometer. We also thank the University of York Technology Facility and Dr. Nathalie Signoret for their expertise and support with panel design, as well as access to the BD FACSDiscover S8. We would like to thank Prof. David Kent for the use of reagents, and Dr. David Boucher and Dr. Sinisa Savic for providing access to healthy control samples and samples from patients with autoinflammatory disease, respectively. J.L.B. is funded by the MRC National Mouse Genetics Network (MC_PC_21043). J.A., I.S.H., and J.L.B. are funded by the 10.13039/501100000272National Institute for Health and Care Research (NIHR) Leeds 10.13039/100014461Biomedical Research Centre (BRC) (NIHR203331). I.S.H. is additionally funded by 10.13039/501100015570Blood Cancer UK (22001). The views expressed are those of the authors and not necessarily those of the NIHR or the 10.13039/501100000276Department of Health and Social Care. We further acknowledge VISFO and Max Noble and Fran Pearson for their financial support and valuable input into panel design, respectively. The graphical abstract/Figures were created using BioRender.

## Author contributions

J.A. designed and performed experiments and wrote the manuscript. S.K.M. and K.G.H. performed experiments, provided advice on experimental design, and provided comments on the manuscript. S.S. provided advice on experimental design and R.L., A.C.R., R.M.D.T., C.C., and I.S.H. supervised the project and provided advice on experimental design and interpretation. J.L.B. supervised the project, designed experiments, and co-wrote the manuscript.

## Declaration of interests

J.L.B. has grant funding from Farnia Biologics and I.S.H. has grant funding from Incyte and Atavistik Bio.
